# A study on the correlation of the asymmetric regulation between the periaqueductal gray and the bilateral trigeminal nucleus caudalis in migraine male rats

**DOI:** 10.1186/s10194-023-01559-4

**Published:** 2023-03-20

**Authors:** Zhijian Cao, Wenjing Yu, Luping Zhang, Jiajia Yang, Jiafei Lou, Maosheng Xu, Zhengxiang Zhang

**Affiliations:** 1 The First School of Clinical Medicine of Zhejiang Chinese Medical University, Hangzhou, China; 2 Department of Radiology, The First Affiliated Hospital of Zhejiang Chinese Medical University (Zhejiang Provincial Hospital of Traditional Chinese Medicine), 54 Youdian Road, Hangzhou, China; 3 Department of Radiology, Hangzhou TCM Hospital Affiliated to Zhejiang Chinese Medical University, Hangzhou, China; 4 Department of Neurology, The First Affiliated Hospital of Zhejiang Chinese Medical University (Zhejiang Provincial Hospital of Traditional Chinese Medicine) Key Laboratory of Neuropharmacology and Translational Medicine of Zhejiang Province, 54 Youdian Road, Hangzhou, China

**Keywords:** Migraine, Magnetic resonance spectroscopy, Trigeminal nucleus Caudalis, Asymmetric regulation

## Abstract

**Background:**

The study was designed to explore the correlation of the asymmetric regulation between periaqueductal gray (PAG) and bilateral trigeminal nucleus caudalis (TNC) in migraine rats through studying the changes of metabolites in pain regulatory pathway of acute migraine attack.

**Methods:**

Thirty male Sprague–Dawley (SD) rats were randomly divided into three groups: blank, control, model groups. Then, blank group was intraperitoneally injected with ultrapure water, while control group injected with saline and model group injected with Glyceryl Trinitrate (GTN). Two hours later, PAG and bilateral TNC were removed respectively, and metabolite concentrations of PAG, Left-TNC, Right-TNC were obtained. Lastly, the differences of metabolite among three brain tissues were compared.

**Results:**

The relative concentrations of rNAA, rGlu, rGln, rTau, rMI in PAG or bilateral TNC had interaction effects between groups and sites. The concentration of rLac of three brain tissues increased in migraine rats, however, the rLac of LTNC and RTNC increased more than that of PAG. Besides, the concentrations of rNAA and rGln increased in RTNC, while rGABA decreased in RTNC.

**Conclusions:**

There is correlation between PAG, LTNC and RTNC in regulation of pain during acute migraine attack, and the regulation of LTNC and RTNC on pain is asymmetric.

## Background

Migraine is a debilitating neurological disorder that leads to reduce the quality of life, increase anxiety and depression, and increase brain infarction and cognitive dementia, affecting an estimated 100 million people worldwide [1, 2]. With the increase of the global population, the prevalence of migraine is also on the rise which increases the burden of the health, living and economic worldwide [3]. However, the pathogenesis of migraine is complex and needs to be further studied. At present, trigeminal vascular reflex theory is believed to be the core mechanism of migraine, the central projections of injurious primary afferent fibers such as dura enter the caudal canal of medulla oblongata in the brainstem through the trigeminal tract, terminating mainly in the trigeminal nucleus caudalis (TNC), so the inhibition of TNC has been shown to be a predictor of efficacy in the treatment of migraine [4–7]. The research on TNC is mainly conducted on both sides. However, with the in-depth research on the left and right brain functions, it is found that the two hemispheres have different functions [8], at the same time, previous studies [9] have found that after electroacupuncture stimulation of the left and right thalamus in the treatment of migraine, the values of NAA /Cr and Cho/Cr in the left thalamus are significantly increased compared with those before treatment. In addition, there is almost no statistical difference in Cho/ Cr while the values of NAA/ Cr in the right thalamus increased compared with those before treatment. This result suggests that the pathogenesis of migraine may also have differences between left and right regulatory pathways.

The brainstem pain regulatory system plays an important role in the pathophysiology of migraine by affecting trigeminal vascular nociceptive information transmission and central sensitization [10]. Current studies [11, 12] have found that the pain regulation system in the descending brainstem is mainly from the periaqueductal gray (PAG) -rostral ventromedial medulla (RVM) circuit to the medullary and spinal dorsal horn, and the pain regulation system can play a bidirectional role resulting in the suppression and facilitation of pain. Among them, PAG is the core part of the central endogenous analgesic system, which can inhibit the nociceptive afferents of the trigeminal neurovascular system to exert analgesic function.

Proton magnetic resonance spectroscopy (^1^H-MRS) is one of the methods for noninvasive quantitative analysis of metabolism, biochemical changes and compounds in human organs and tissues. Many metabolites such as N-acetyl aspartate (NAA), lactate (Lac), creatine (Cr) and glutamate (Glu) can be detected simultaneously [13]. In recent years, MRS has been used to study the changes of brain metabolites and neurotransmitters in migraine patients [14–16]. Therefore, in this study, MRS was used to observe the changes of metabolism and neurotransmitters of bilateral TNC and PAG through Glyceryl Trinitrate (GTN) -induced migraine rat model, and the relationship between the differences of metabolism and neurotransmitter changes during pain attack was compared, so as to reveal the correlation between the asymmetric regulation of PAG and bilateral TNC in migraine. Lastly, previous studies [17–19] explored the correlation between GTN-induced hyperalgesia and temporal changes in rats, and found that GTN concentration reached its maximum in the brain 2 hours after GTN injection, causing a significant increase in dural mast cell degranulation and an upregulation of mRNA and protein expression of the nociceptive marker c-fos in TNC; On this basis, this study selected 2 h after rat modeling to study the changes of metabolites.

## Materials and methods

### Animals

Thirty male Sprague–Dawley rats, weighing 140-170 g, purchased from Shanghai Xipuer-Bikai Laboratory Animal Co., Ltd. (License No. SCXK [hu] 2013–0016) were used in the study. The rats were housed in the Laboratory Animal Center of Zhejiang Chinese Medical University with a 12-h light–dark cycle (22.7 ± 0.2 °C and 49 ± 10% humidity) and (had) free access to water and food. The study was approved by the Laboratory Animal Center of Zhejiang Chinese Medical University. Besides, animal feeding management and animal experiment operation shall comply with《Zhejiang province Administration Rule of Laboratory Animal》.

### Experimental design

Thirty rats were weighed and numbered, then the rats were randomly divided into 3 groups by the excel (*n* = 10/group): blank group, control group and model group. All of the three groups were gavaged with ultra-pure water (administration volume 1 ml/100 g) for 7 consecutive days, once a day in the morning. At the end of gavage on the seventh day, after 0.5 hours of adaptation in a transparent tempered glass box (30 cm × 30 cm × 40 cm in length, width and height), the blank group was intraperitoneally injected with ultra-pure water (administration volume: 0.2 ml/100 g), the control group was intraperitoneally injected with normal saline (administration volume: 0.2 ml/100 g), and the model group was intraperitoneally injected with GTN (administration dose: 10 mg/kg, administration volume: 0.2 ml / 100 g). Molding time: 9:30–10:00 a.m. After intraperitoneal injection, the rats were placed in the transparent tempered glass box, and the surrounding environment was kept quiet. All the animals were recorded for 2 hours of behavioral science. Within 2 h, the model animals showed burnout and reduced movement.

### Samples collection

Two hours after modeling, the rats were completely anesthetized by intraperitoneal injection of 1% sodium pentobarbital (administration dose: 50 mg/kg, administration volume: 0.5 ml/100 g). The whole brain tissue removed from the rat was immediately stored in an ultra-low temperature refrigerator at − 80 °C. Then, the brain tissues of the PAG, LTNC and RTNC were respectively obtained according to the Stereoscopic Map of Rat Brain in the frozen microsection machine at − 20 °C and stored in the refrigerator at − 80 °C.

### ^1^H-MRS analysis

PAG/LTNC/RTNC samples were damaged in one rat in the blank group. Finally, 87 samples were included in the study. The MRS of all the samples was detected in College of Life Sciences, Zijingang Campus, Zhejiang University. Firstly, all the samples were weighed with an electronic balance and recorded. Secondly, the samples were individually ground in a grinding tube containing 600ul of 0.05% TSP-d4 heavy water and then transferred to a specific 5-mm diameter NMR tube. Lastly, all the samples were analyzed on a Bruker 600 MHz AVANCE III spectrometer equipped with a 5 mm-BBFO probe. After D2O locking field and automatic homogenization field, the pressure water peak map was obtained by NoesyPR1D sequence with the acquisition time of 1.7 s, the acquisition times of 128, the data points of 32 K, and the scanning time of 8.9 min. In the end, the original data was obtained by using Bruker Topspin 3.2.

### ^1^H-MRS data processing

**Fig. 1:**
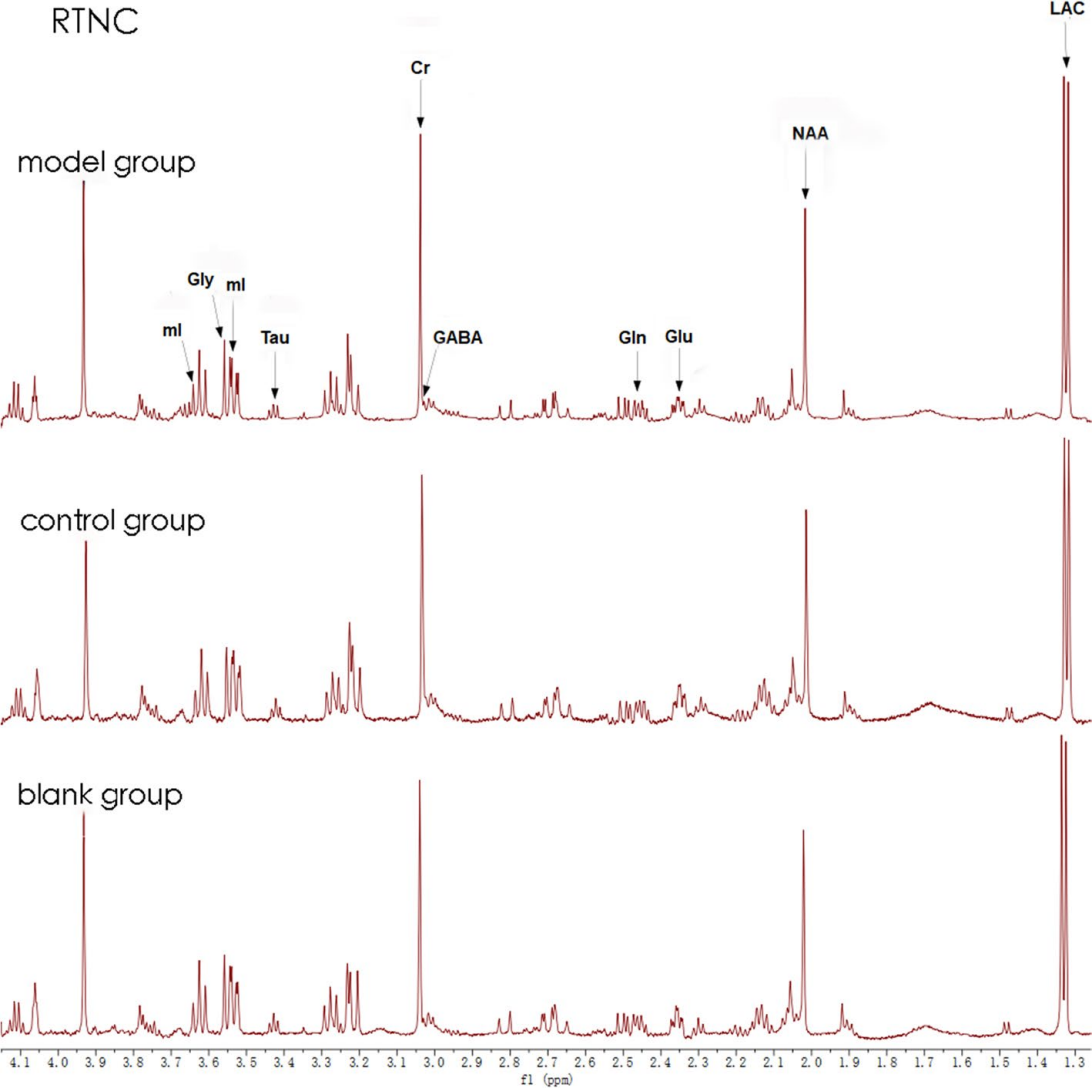
RTNC MRS image of the three groups sample graph, blank (14 mg), control9 (14 mg), model (18 mg)

All the free induction decays (FIDs) from ^1^H-MRS of the brain tissues were processed by MestRe Nove, a spectrum analysis and processing software, via fourier transformation, phase correction, and baseline correction with trimethylsilane propionate (TSP) as internal standard (chemical shift 0.00 PPM). The relevant metabolite concentrations were calculated by the formula CM = AM/ATSP*fP*CTSP*MWM/WM and denoted by “i”; The ratio of the relevant metabolite concentration to TSP and weight (i/TSP/weight) is considered as the absolute concentration and is denoted by “c”; The ratio of the relevant metabolite concentration to creatine phosphate (tCr) is considered as the relative concentration and is denoted by “r” (Fig. 1).

### Statistical analysis

Statistical analyses were performed with SPSS Statistics (version 25.0). GraphPad Prism(version 9.0) statistical graphics. The measurement data were expressed as mean ± standard deviation (^−^x ± s) if they were in accordance with normal distribution, otherwise, they were expressed as median (P25, P75). Enumeration data were expressed as rate or constituent ratio. The differences of brain metabolites in PAG, RTNG, LTNG of the three groups were analyzed by generalized estimating equation. For brain metabolites with interaction effects, it is necessary to fix the location and analyze the differences between groups in each location separately. In addition to that, for the data with normal distribution and homogeneity of variance, one-way analysis of variance was used for comparison between multiple groups (more than 2 groups), and LSD method was used for pairwise comparison between groups. For the data with were non-normal, Kruskal-Wallis H method was used for comparison between multiple groups, and all pairwise method was used for pairwise comparison between groups. The value of *P* < 0.05 was considered statistically significant.

## Results

### The interaction analysis of brain metabolites in PAG, LTNC and RTNC

We found that the group and site interaction effects of brain metabolites of rNAA (*P* = 0.036), rGlu (*P* = 0.001), rGln (*P* = 0.001), rTau (*P* = 0.023), rMl (*P* = 0.023) were statistically different, while the other brain metabolites (rLac, rAla, rAce, rGABA, rGly) group and site interaction effects were not statistically significant.

**Fig. 2:**
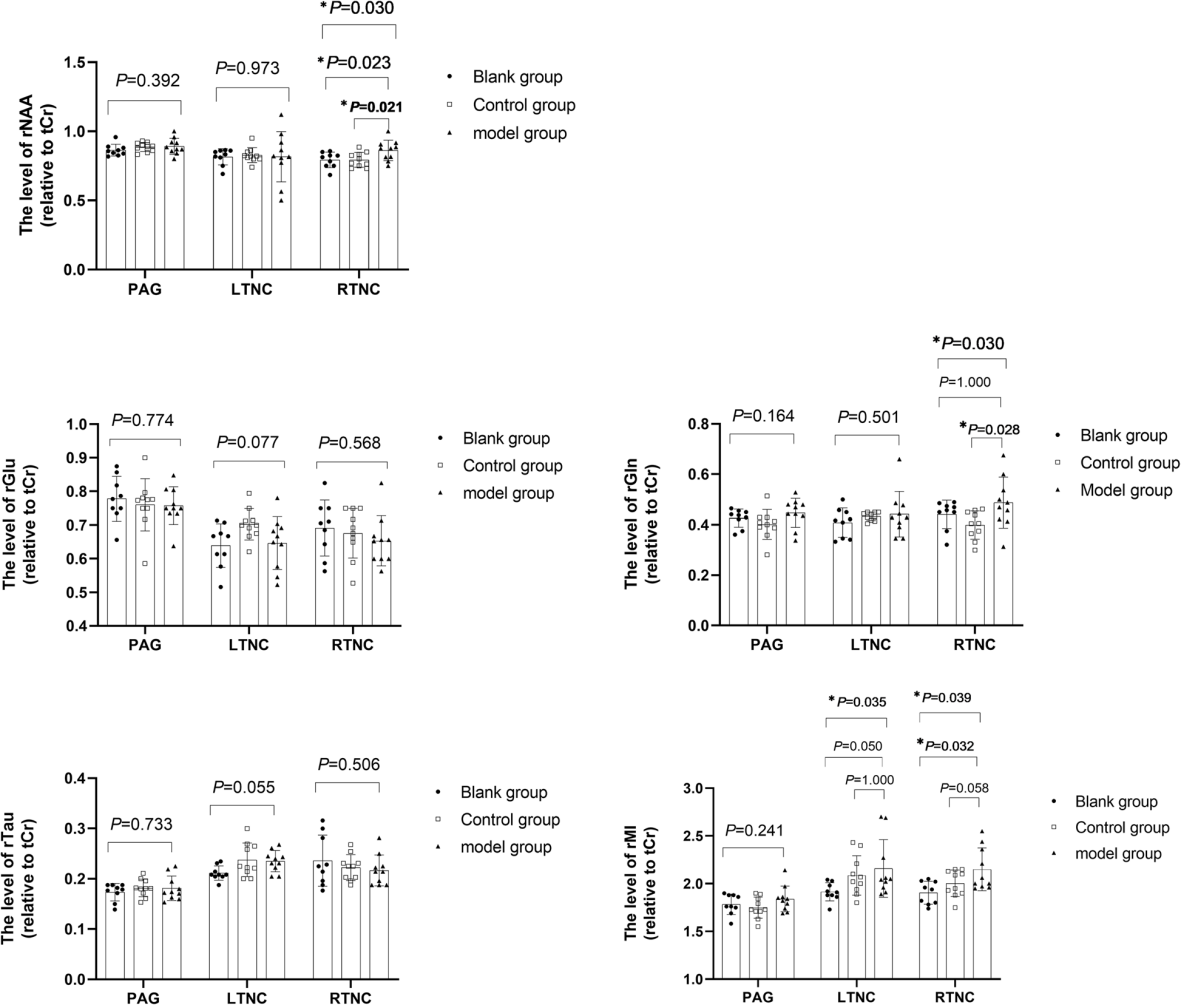
Comparison of rNAA, rGln, rGlu, rTau and rMl in PAG, LTNC and RTNC (*n* = 29); * denotes *P* < 0.05, ** denotes *P* < 0.01; The rNAA, rGln of RTNC in the control group were lower than the model group, while the rGlu, rTau of RTNC had no significant difference between groups

As shown in Fig. 2, there was no significant difference of rNAA in PAG and LTNC between groups (*P* > 0.05). However, the rNAA of control group (*P* = 0.021) and blank group (*P* = 0.023) were lower than those of model group in RTNC.

Furthermore, there was no significant difference of rGln in PAG and LNTC between groups (*P* > 0.05). However, the rGln of the control group (*P* = 0.028) was lower than that of the model group, and there was no significant difference between the blank group and the model group (*p* > 0.05) in RTNC.

Besides, there was no significant difference of rGlu in PAG，LTNC and RTNC between groups (*P* > 0.05). Likewise, there was no significant difference of rTau in PAG, LTNC and RTNC between groups (*P* > 0.05).

Lastly, in the Fig. 2, we can know that there was no significant difference of rMI in PAG between groups (*P* > 0.05). However, there was significant difference between LTNC rMl groups (*P* = 0.035) which suggested that there was no significant difference between control group and model group (*P* = 1.000), and no significant difference between blank group and model group (*P* = 0.050). There was significant difference between RTNC rMl groups (*P* = 0.039) which showed that there was no significant difference between control group and model group (*P* > 0.05), and significant difference between blank group and model group (*P* = 0.032).

### Comparison of relative concentration of total brain metabolites in different parts

**Fig. 3:**
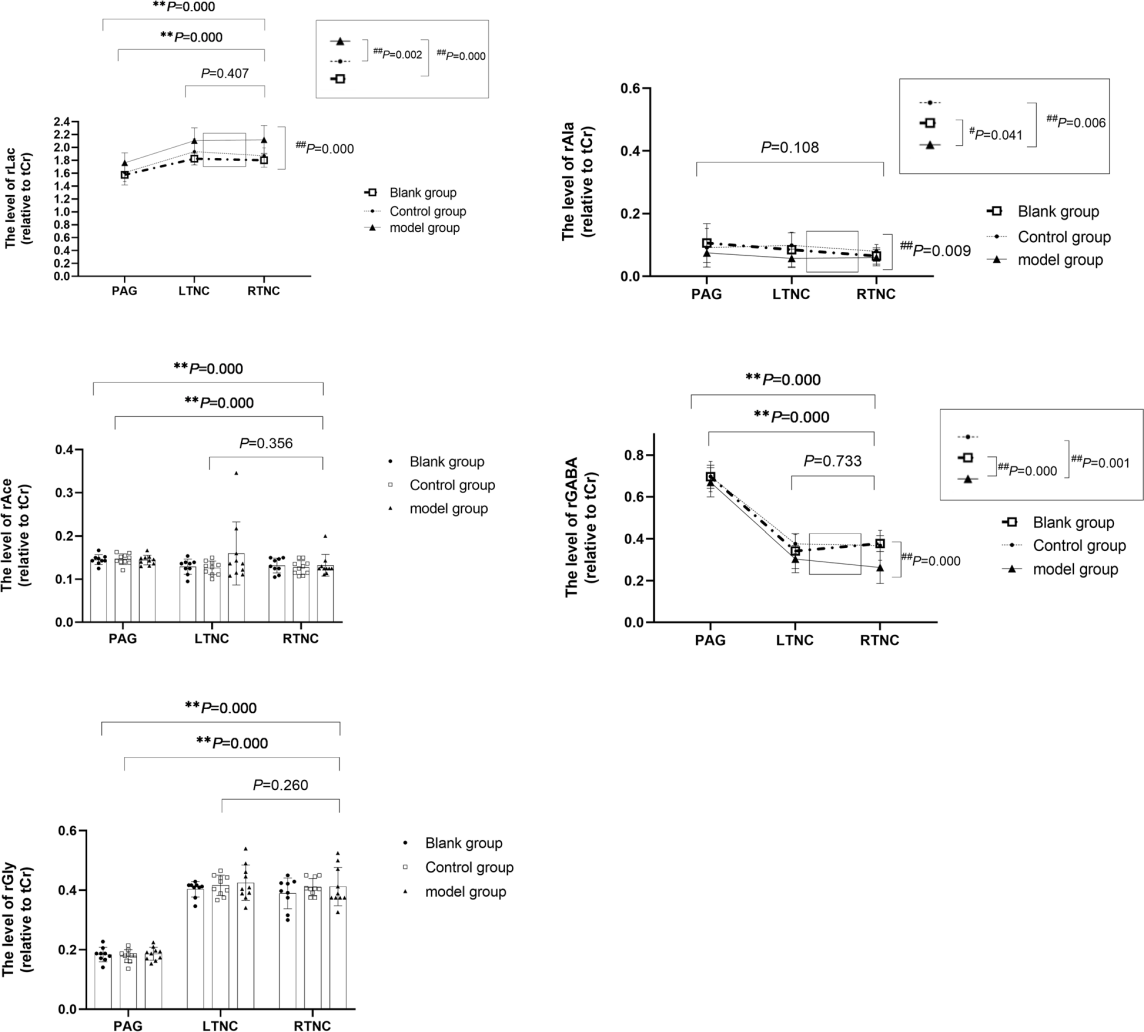
Comparison of relative concentration of total brain metabolites in different parts. Compared with RTNC, * denotes *P* < 0.05, ** denotes *P* < 0.01; Compared with the model group, # indicates *P* < 0.05, and ## indicates *P* < 0.01. There were no significant differences in rLac, rAla, rAce, rGABA and rGly between LTNC and RTNC; There were significant differences in rLac, rAce, rGABA and rGly between PAG and RTNC which showed the rLac and rGly of PAG were lower than those of RTNC while the rAce and rGABA of PAG were higher than those of RTNC. The rLac, rAla and rGABA were statistically different among the groups (PAG, LTNC and RTNC), indicating the rAla and rGABA in the model group were lower than those in the control group and the blank group, when the rLac in the model group were higher than those in the control group and the blank group

As shown in the Fig. 3, there were significant differences of rLac between groups (*P* = 0.000) which showed that the differences between blank group (*P* = 0.000), control group (*P* = 0.002) and model group were statistically significant. In addition to that, there were statistically significant differences of rLac between PAG, LTNC and RTNC (*P* = 0.000), suggesting the rLac difference between PAG and RTNC was statistically significant (*P* = 0.000), while the rLac difference between LTNC and RTNC was not statistically significant (*P* > 0.05).

There were significant differences of rAla between groups (*P* = 0.009) which showed that the differences between blank group (*P* = 0.041), control group (*P* = 0.006) and model group were statistically significant, on the contrary, there were no statistically significant differences of rAla between PAG, LTNC and RTNC (*P* > 0.05).

About the rAce, there were no significant differences between groups (*P* > 0.05). However, there were statistically significant differences between PAG, LTNC and RTNC (*P* = 0.000), suggesting the difference between PAG and RTNC was statistically significant (*P* = 0.000), while the difference between LTNC and RTNC was not statistically significant (*P* > 0.05).

Moreover, there were significant differences of rGABA between groups (*P* = 0.000) which showed that the differences between blank group (*P* = 0.000), control group (*P* = 0.001) and model group were statistically significant, likewise, there were statistically significant differences of rGABA between PAG, LTNC and RTNC (*P* = 0.000), suggesting the difference between PAG and RTNC was statistically significant (*P* = 0.000), while the difference between LTNC and RTNC was not statistically significant (*P* > 0.05).

Finally, about the rGly, there were no significant differences between groups (*P* > 0.05). However, there were statistically significant differences between PAG, LTNC and RTNC (*P* = 0.000), suggesting the difference between PAG and RTNC was statistically significant (*P* = 0.000), while the difference between LTNC and RTNC was not statistically significant (*P* > 0.05).

### Comparison of brain metabolites in different parts (no interaction effect)

As shown in the Tables 1 and 2. In the PAG, the differences of rLac between groups were statistically significant (*P* = 0.026) which showed the model group was significantly higher than the blank group (*P* = 0.012) and the control group (*P* = 0.032).

**Table 1:** The comparison of the relative concentration of rLac in different sites

Sites		Blank group	Control group	Model group
	N	9	10	10
PAG	relative concentration(r)	1.574 ± 0.156^#^	1.609 ± 0.141^#^	1.762 ± 0.156
	F		4.237	
	P1		0.026	
	P	0.012	0.032	
LTNC	relative concentration(r)	1.824 ± 0.096^##^	1.934 ± 0.200^#^	2.106 ± 0.197
	F		6.355	
	P1		0.006	
	P	0.002	0.037	
RTNC	relative concentration(r)	1.800 ± 0.107^##^	1.872 ± 0.121^##^	2.119 ± 0.223
	F		10.510	
	P1		0.000	
	P	0.000	0.002	

Notice: ^#^Represents comparison with the model group, *p* < 0.05; ^##^Represents comparison with the model group, *p* < 0.01; P1 Indicates the *p* value of one-way analysis or Kruskal-Wallis H test

**Table 2:** The comparison of different metabolites on RTNC

Brain metabolites	groups	N	RTNC			
			relative concentration(r)	F	*P*1	*P*
rNAA	blank group	9	0.793 ± 0.056#	4.015	0.030	0.023
	control group	10	0.794 ± 0.053^#^			0.021
	model group	10	0.862 ± 0.073			
rGABA	blank group	9	0.377 ± 0.038^##^	9.321	0.001	0.001
	control group	10	0.368 ± 0.071^##^			0.001
	model group	10	0.263 ± 0.077			
rGln	blank group	9	0.457(0.413,0.479)		0.030	1.000
	control group	10	0.399 ± 0.055^#^			0.028
	model group	10	0.487 ± 0.101			

Notice: ^#^Represents comparison with the model group, *p* < 0.05; ^##^Represents comparison with the model group, *p* < 0.01; P1 Indicates the p value of one-way analysis or Kruskal-Wallis H test

In the LTNC, the differences of rLac between groups were statistically significant (*P* = 0.006) which showed the model group was significantly higher than the blank group (*P* = 0.002) and the control group (*P* = 0.037).

In the RTNC, the differences of rLac between groups were statistically significant (*P* = 0.000) which showed the model group was significantly higher than the blank group (*P* = 0.000) and the control group (*P* = 0.002).

In the RTNC, the differences of rNAA between groups were statistically significant (*P* = 0.030) which showed the model group was higher than that of the blank group (*P* = 0.023) and the control group (*P* = 0.021).

In the RTNC, the differences of rGABA between groups were statistically significant (*P* = 0.001) which showed the model group was lower than that of the blank group (*P* = 0.001) and the control group (*P* = 0.001).

In the RTNC, the differences of rGln between groups were statistically significant (*P* = 0.030) which showed the model group was higher than that of the control group (*P* = 0.028). However, there was no statistical difference between the model group and the blank group (*P* = 1.000).

## Discussion

The current study was designed to explore the correlation of the asymmetric regulation between the periaqueductal gray (PAG) and the bilateral trigeminal nucleus caudalis (TNC) in migraine rats through the changes of metabolites and neurotransmitters of PAG and TNC in the pain regulatory pathway of acute migraine attack. In our study, we found that the metabolites and neurotransmitters of the PAG, the LTNC and the RTNC had interaction effects between groups and sites. The concentration of the rLac of the PAG, LTNC and RTNC increased in migraine rats, however, the rLac of the LTNC and the RTNC increased more than that of the PAG. Besides, the concentrations of metabolites and neurotransmitters were asymmetrical in the LTNC and the RTNC which showed that the rNAA and rGln increased in the RTNC, while rGABA decreased in the RTNC. Therefore, we can conclude that there is a correlation between the PAG, LTNC and RTNC in the regulation of pain during the acute attack of migraine, and the regulation of the LTNC and RTNC on pain is asymmetric.

Our study found that the rLac increased in PAG, RTNC, and LTNC in migraine rats. Lac is the product of anaerobic glycolysis. The increase of Lac in the brain is thought to be caused by mitochondrial dysfunction or insufficient oxygen content [20]. However, in recent years, it has been found that increased neuronal activity can lead to a rapid and transient increase of Lac in brain [21]. In addition, it has also been found that Lac increases in the occipital cortex and cerebellum during the interval of migraine attacks [22–25]. TNC is an important structure for nociceptive generation, so when pain stimuli are transmitted to TNC, the neuronal activity of TNC increases, which leads to the increase of Lac in TNC on both sides, suggesting that TNC neurons are activated bilaterally. PAG is the core part of the central endogenous analgesic system, in the early stage of migraine attack, the Lac of PAG increased less than that of TNC which suggests that the neuronal activity of PAG also increased less than that of TNC, indicating that PAG neurons respond to pain in the early stage, however, it needs further investigation whether this early response promotes pain or involved in pain suppression.

Our study also found that rNAA increased on RTNC in migraine rats, whereas rNAA did not show such a difference on LTNC. NAA is synthesized by aspartic acid and acetyl-CoA (CoA) under the catalysis of aspartic acid N-acetyltransferase [26], which is mainly concentrated in gray matter rich areas in the brain [27]. The synthesis of NAA depends on the integrity of neuronal mitochondria, and the fluctuation of concentration of NAA may occur simultaneously with the change of adenosine triphosphate (ATP) which suggests that it is closely related to metabolic energy [28, 29]. As a result, when neurons are stimulated, NAA will be released [30]. TNC is an important structure for nociceptive production, which contains a relatively rich ratio of neurons to glial cells, during pain stimulation, neurons release a large amount of stored NAA, which increases its concentration transiently. However, in our study, there is no similar phenomenon on LTNC and PAG which is an interesting phenomenon, According to Lac, we can know that bilateral TNC and PAG have activation changes in the early stage of migraine, but NAA, as a marker of neuronal activity, only increases in RTNC, therefore, it needs further study whether NAA is an important marker of neuronal activation related to pain generation or transmission. At the same time, this phenomenon also suggests that TNC on both sides have differences in conduction of pain sensation when exposed to pain stimuli.

In our study, we also found that rGABA decreased and Gln increased in RTNC of migraine rats. Gln is a metabolite of the excitatory neurotransmitter glutamate (Glu), specifically produced in brain astrocytes by glutamate and ammonia under the catalysis of glutamine synthetase. Then, glutamine is transported back to neurons, where it is converted to Glu by mitochondrial glutaminase [31]. This circulation is thought to prevent neuroexcitability caused by the production of too much Glu in the brain. GABA is a major inhibitory neurotransmitter in the central nervous system, which is directly synthesized by Glu through glutamate decarboxylase [32]. Meanwhile, the imbalance of Glu-GABA concentration has been considered as another hypothesis for the occurrence of migraine [33]. Zhe Ma et al. [18] found a significant increase of Glu/GABA+ in the thalamus of migraine rats, whereas Nastaren Abad et al. [19] did not find the similar increase in cortical regions of the migraine rat. The increased Gln in our study may be due to the rapid action of the excitatory neurotransmitter Glu early in migraine attack, causing pain to occur and producing excessive Gln. At the same time, when the pain stimulus is transmitted to the RTNC, the inhibitory neurotransmitter GABA decreases which reduces the pain threshold and improves the excitability of RTNC, and thus accelerates the conduction of pain stimulus signal.

In addition, our study found that the relative concentrations of brain metabolites rNAA, rGlu, rGln and rTau in the PAG and the bilateral TNC had interaction effects between groups and sites. However, the interaction effect could not be proved by current experiments and its significance needs to be further studied.

Our study identified asymmetric changes of metabolic substances and neurotransmitters in the left and right TNC during headache attack in a migraine rat model, which was first found in the study of migraine attack mechanism. These asymmetrical changes are consistent with our recent clinical findings which is that some migraine patients with patent foramen ovale (PFO) often have long-term unilateral headache, in recent years, many researches [34–36] have identified a relationship between PFO and migraine headache which also indirectly suggests the possibility of bilateral asymmetric regulation of migraine. In addition to that, Momoh-Ojewuyi et al. [37] found that a small percentage of people experienced long-term migraine with unilateral fixation, which is called side locked unilateral headache (SLUH). Besides, Shinoura N et al. [38] found that stress-related vascular responses were suppressed in the right cerebral hemisphere during migraine interictal periods, which indirectly suggested left-right cerebral functional asymmetry in migraine mechanisms. In addition, this functional asymmetry has been studied in other disorders, for example, antidepressants can increase activity in the left hemisphere [39, 40], Kong XZ et al. [41] have also shown that autism spectrum disorder (ASD) is associated with a subtle reduction in cortical thickness asymmetry that is widely distributed in cortical regions. Therefore, asymmetric regulation of brain function has been gradually discovered and studied in some diseases. Finally, our study points out that there are asymmetric changes of metabolites in left and right TNC during migraine attacks, these asymmetrical changes are consistent with Momoh-Ojewuyi et al’s found [37] which not only provides a new insight into the clinical application of unilateral nerve electrical stimulation in the treatment of migraine when drugs cannot provide sufficient relief, but also provides a new direction for future research on the pathogenesis of migraine.

In this study, the rat model of migraine was induced by GTN which mimic those migraine patients caused by meningeal inflammation and dural mast cell degranulation in clinic [18]. Although some migraine rats in the model group showed some complications such as burnout behavior at the end of the experiment, this model was preferred compared to other migraine rat models because of the other rat migraine models [42, 43] require invasive manipulation of the rat head, which can affect the MRI scan. Therefore, in order to obtain a more stable and undisturbed spectrum, we chose GTN for migraine induction.

The behavioral manifestations of migraine model induced by nitroglycerin were mostly as follows: allodynia [44] and hyperalgesia [45]. Allodynia test include heat sensitivity and Mechanical nociceptive thresholds, both are hurtful stimuli. GTN-induced hyperalgesia included the tail flick test and the formalin test, which is also the hurtful stimuli. These behavioral tests affect the purpose of the experiment. For another, the spontaneous behavior of headache induced by GTN was unstable. Although light sensitivity changes before and after the model, it does not have repeatability. Burrowing behavior, running wheel activity and light sensitivity cannot be used to establish successful behavioral criteria for GTN mode [46]. Recent studies have shown that reflex blink-associated EMG response [47] can be used as a successful evaluation criterion for 2 h modeling of migraine induced by GTN, but this test requires anesthesia rather than wakefulness, so it is not suitable for this study.

## Conclusion

In this study, we found that both TNC and PAG were activated in the early stage of migraine, but there were differences in the pain response of the left and right TNC, and the right TNC played a key role in the generation and conduction of pain, which also suggested that the occurrence of migraine was asymmetric regulation of TNC on both sides. And the role of early activation of PAG in migraine regulation needs to be further studied.

In conclusion, we found the differences in the concentrations of metabolic substances between PAG and TNC in the left and right sides of migraine rats, revealing the asymmetry of TNC in the regulation of migraine, and PAG is also activated in the early stage of migraine, which provides theoretical help for further understanding the pathophysiology basis of migraine.

However, this study has some limitations: Firstly, this study only compared the changes of metabolites in brain tissue 2 h after modeling, so subsequent studies need to select multiple time points for measurement to conduct more in-depth research; Secondly, male rats were used in this study, and female rats need to be included in subsequent experiments which could make the experimental design more perfect. Thus, more researches are also necessary.

## Data Availability

The datasets used and/or analysed during the current study are available from the corresponding author on reasonable request.

## Abbreviations

PAG: Periaqueductal gray
SD: Sprague–Dawley
NAA: N-acetylaspartate
Gln: Glutamine
MI: Myo-inositol
GABA: γ-aminobutyric acid
Cho: Choline
^1^H-MRS: Proton magnetic resonance spectroscopy
Ala: Aminolevulinic acid
Gly: Glycine
PFO: Patent foramen ovale
ASD: Autism spectrum disorder
TNC: Trigeminal nucleus caudalis
GTN: Glyceryl Trinitrate
Glu: Glutamate
Tau: Taurine
Lac: Lactate
Cr: Creatine
RVM: Rostral ventromedial medulla
Ace: Angiotensin-Converting Enzyme
ATP: Adenosine triphosphate
CGRP: Calcitonin Gene-Related Peptide
